# The Impact of Refined, Bleached, and Deodorized Palm Oil and Sunflower Lecithin on the Physicochemical Properties and Oxidative Stability of Egg-Free Mayonnaise

**DOI:** 10.1021/acsomega.6c00909

**Published:** 2026-06-09

**Authors:** Shin-Yong Yeoh, Nur Iesya Safiah Abdul Hamid, Lubowa Muhammad, Ahmad Syahir Zulkipli, Faheem Ullah, Hui-Ling Tan, Thuan-Chew Tan, Azhat Mat Easa

**Affiliations:** † Food Technology Division, School of Industrial Technology, 26689 Universiti Sains Malaysia, Building G07, Persiaran Sains, Minden Heights, Pulau Pinang 11800, Malaysia; ‡ Department of Food Innovation and Nutrition, Faculty of Agriculture and Environmental Sciences, 187215 Mountains of the Moon University, P.O. Box 837, Fort Portal, Uganda; § Earth Material Characterization Laboratory, Centre for Global Archaeological Research, Universiti Sains Malaysia, Minden Heights, Pulau Pinang 11800, Malaysia; ∥ Bioresource Technology Division, School of Industrial Technology, Universiti Sains Malaysia, Building G07, Persiaran Sains, Minden Heights, Pulau Pinang 11800, Malaysia; ⊥ School of Science, 65210 Monash University Malaysia, Jalan Lagoon Selatan, Bandar Sunway, Selangor Darul Ehsan 47500, Malaysia

## Abstract

This study investigated the potential of refined, bleached, and deodorized palm oil (RBD PO) and sunflower lecithin (SL) as egg-yolk replacers in egg-free mayonnaise. Formulations containing RBD PO (5–15%) and SL (1–3%), individually and in combination, were evaluated for physicochemical, textural, microstructural, and oxidative properties. No significant differences in pH or water activity were observed (*p* > 0.05). RBD PO increased firmness and reduced cohesiveness, whereas SL enhanced viscosity and emulsion stability by promoting the formation of smaller and more uniformly distributed oil droplets. Microstructural analysis showed that excessive RBD PO led to droplet aggregation, while SL improved interfacial stability. SL-containing formulations exhibited lower peroxide and p-anisidine values during storage, indicating enhanced oxidative stability. Among the formulations tested, PO5SL3 showed the optimal combination of structural integrity, emulsion stability, and oxidative resistance, approaching the functional and stability properties of conventional egg-based mayonnaise. These findings demonstrate that emulsion behavior is governed by lipid composition and phospholipid-mediated interfacial stabilization. The combined use of RBD PO and SL enables the development of stable egg-free mayonnaise with controlled texture and oxidation behavior, highlighting their potential as functional egg-yolk alternatives in structured plant-based emulsions.

## Graphical abstract

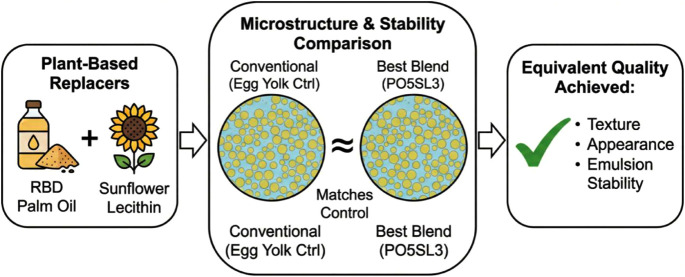

## 1 Introduction

Mayonnaise is a popular sauce with a thick, semisolid oil-in-water emulsion, containing 65–80% fat, where product quality depends critically on interactions between dispersed oil droplets.^1^ It is made by emulsifying edible oil with egg yolk in an aqueous phase containing vinegar, salt, and seasonings.^2^ However, raw egg-based products remain associated with risks of Salmonella contamination, high cholesterol content, and cost constraints.^1,3^ Additionally, fat oxidation during storage compromises shelf life, nutritional quality, and safety.^4^ Furthermore, the global shift toward more sustainable, health-conscious dietary patterns has significantly increased consumer interest in plant-based and vegan food products, driven by environmental and health benefits.^5^ These factors have driven interest in plant-based alternatives with emulsifying, stabilizing, and texturizing properties for the development of egg-free mayonnaise.

Refined, bleached, and deodorized palm oil (RBD PO) represents a promising candidate due to Malaysia’s position as the world’s second-largest producer, its exceptional oxidative stability, mild flavor profile, and cost-effectiveness.^6,7^ Sunflower lecithin (SL), rich in phospholipids including phosphatidylcholine (PC), phosphatidylethanolamine (PE), phosphatidylinositol (PI), and phosphatidic acid (PA), offers superior emulsion-stabilizing capabilities attributed to its lower PC/PE ratio and negative spontaneous curvature at interfaces.^8^ As a non-GMO, hypoallergenic emulsifier with antioxidant activity, SL aligns with broader market trends, wherein global lecithin demand is rising by about 7% per annum between 2018 and 2023.^9,10^

While virgin red palm oil has been reported to enhance certain physical attributes of mayonnaise,^11^ and various plant proteins have been explored as egg replacers with mixed effects on texture and sensory quality,^1^ the combined use of RBD PO and SL in egg-free mayonnaise remains largely unexplored. This represents a critical knowledge gap, as the high oxidative stability and saturated triacylglycerol content of RBD PO may complement the interfacial and emulsifying functionality of phospholipid-rich SL, offering a promising strategy for developing stable plant-based emulsions.

Most existing studies on egg-free mayonnaise focus primarily on ingredient substitution or formulation optimization, with limited mechanistic insight into how oil composition and emulsifier chemistry jointly govern emulsion microstructure, oxidative behavior, and textural performance. In particular, the interactions between saturated lipid systems and phospholipid-based emulsifiers in concentrated food emulsions remain poorly understood, despite their critical role in controlling droplet organization, rheology, and stability.

From a physicochemical perspective, emulsion stability is governed by interfacial phenomena, including emulsifier adsorption, interfacial film elasticity, and lipid phase behavior. The crystallization behavior of saturated triacylglycerols and their interaction with phospholipid-based emulsifiers strongly influence droplet organization, rheological properties, and oxidative stability.^12^ However, these interfacial mechanisms have not been systematically examined in egg-free mayonnaise systems formulated with refined palm oil and lecithin. Therefore, this study aims to investigate the effects of replacing egg yolk with RBD PO and SL on the physicochemical, microstructural, and oxidative properties of mayonnaise. Specifically, the work examines how the interplay between lipid composition and emulsifier functionality influences pH, viscosity, color, emulsion stability, droplet characteristics, and lipid oxidation behavior. It is hypothesized that combining RBD PO and SL can replicate key emulsifying functions of egg yolk, producing an egg-free mayonnaise with comparable structural integrity, improved oxidative stability, and suitability for clean-label formulations.

## 2 Materials and Methods

Sunflower oil (Naturel), eggs (Qplus), white vinegar (Yeo’s), potato, superfine sugar (MSM Prai Berhad), and salt (Lotus’s) were purchased from Lotus’s Store Sdn. Bhd. (George Town, Malaysia). SL (19% PC, 7% PE, and 12% PI) was sourced from NOW Foods (Illinois, USA), and RBD PO was obtained from IKO Nature Sdn. Bhd. (Seremban, Malaysia).

### 2.1 Preparation of Mayonnaise

Mayonnaise samples were prepared as outlined in Table 1. The control formulation comprised sunflower oil, boiled potato, sugar, salt, vinegar, and egg yolk. All ingredients were accurately weighed before mixing. The samples were prepared using a 403HB1 hand blender (600 W, Morphy Richards, London). The egg yolk, boiled potato, sugar, salt, and vinegar were initially blended for 2 min at speed level 1. Subsequently, sunflower oil, RBD PO, and/or SL were gradually added while mixing continuously at the same speed for an additional 5 min. Finally, the mixture was homogenized for an additional 2 min. The prepared samples were then transferred into sterilized containers and stored under refrigeration for further analysis. The ratio of RBD PO was selected based on the study by Wiguna et al. (2023), who incorporated 5, 10, and 15 g of virgin red PO into mayonnaise. The proportion of SL was determined through preliminary trials to optimize texture and emulsion stability.

**Table 1:** Formulation and Designation of Mayonnaise Samples^a^

	Ingredients (g)							
Sample	Sunflower oil	Boiled potato	Vinegar	Sugar	Salt	Egg yolk	RBD PO	SL
Control	40	40	5	2.5	2.5	10	-	-
PO5	45	40	5	2.5	2.5	-	5	-
PO10	40	40	5	2.5	2.5	-	10	-
PO15	35	40	5	2.5	2.5	-	15	-
SL1	49	40	5	2.5	2.5	-	-	1
SL2	48	40	5	2.5	2.5	-	-	2
SL3	47	40	5	2.5	2.5	-	-	3
PO5SL3	42	40	5	2.5	2.5	-	5	3
PO10SL2	38	40	5	2.5	2.5	-	10	2
PO15SL1	36	40	5	2.5	2.5	-	15	1

a “-” denotes the absence of the ingredient in the formulation.

### 2.2 pH Measurement

The pH of the samples was measured using a calibrated pH meter (Mettler Toledo, USA).^13^ The instrument was calibrated before measurement using standard buffer solutions with pH values of 4.01, 7.00, and 9.21. Three replicates were conducted for each sample.

### 2.3 Water Activity Measurement

Water activity was determined at room temperature using a Mettler Toledo water activity meter (New Jersey, USA).^14^ The device was calibrated using distilled water before analysis. Three replicates were conducted for each sample.

### 2.4 Color Analysis

The color of the mayonnaise samples was evaluated using a Konica Minolta CM-3500d spectrophotometer (Konica Minolta, New Jersey, USA). The sample was evenly spread in a glass container, and color measurements were taken at three different points. The average of the three readings was used to determine the colorimetric parameters based on the CIE L*, a*, and b* system (Fernandes and Mellado, 2018). Three replicates were conducted for each sample.

PO5 represents mayonnaise containing 5 g RBD PO; PO10 represents mayonnaise containing 10 g RBD PO; PO15 represents mayonnaise containing 15 g RBD PO; SL1 represents mayonnaise containing 1 g SL; SL2 represents mayonnaise containing 2 g SL; SL3 represents mayonnaise containing 3 g SL; PO5SL3 represents mayonnaise containing 5 g RBD PO and 3 g SL; PO10SL2 represents mayonnaise containing 10 g RBD PO and 2 g SL; PO15SL1 represents mayonnaise containing 15 g RBD PO and 1 g SL.

### 2.5 Texture Profile Analysis (TPA)

Texture attributes, including firmness, consistency, and cohesiveness, were assessed using a Stable Micro Systems TA-XT2 texture analyzer (London, United Kingdom) employing the back-extrusion method, as described in ref 11. Three replicates were conducted for each sample. Samples were placed in cylindrical containers (35 mm height, 60 mm internal diameter) and filled to the 25 mm mark. A 35 mm diameter disc (Stable Micro Systems, UK) was used to compress the samples at a constant speed of 1 mm/s to a depth of 30 mm. The force-time curve generated during compression was used to calculate the texture parameters. Firmness was determined from the maximum force applied. Consistency was evaluated from the area under the positive portion of the curve. Cohesiveness was derived from the maximum negative force, corresponding to the negative peak on the graph during the probe’s return phase.

### 2.6 Viscosity Measurement

The viscosity of the mayonnaise samples was measured using a Brookfield viscometer (Model DV-II+, Brookfield Engineering) at 23 °C.^11^ Spindle No. 3 was operated at a constant speed of 5 rpm to obtain viscosity readings. Three replicates were conducted for each sample.

### 2.7 Determination of Emulsification Stability

Emulsification stability was evaluated by measuring the creaming index (CI) under both room temperature and thermal conditions.^15^ This method assesses the creaming behavior of oil droplets by quantifying their upward migration within the emulsion. Three replicates were conducted for each sample. Approximately 10 ± 1 mL of the mayonnaise sample was carefully transferred into a cylindrical plastic tube and centrifuged at 3,500 rpm for 30 min. The CI was calculated using the following equation:

$$\%H=\frac{H}{H_{O}}\times 100\%$$

where H is the height of the separated cream layer, and H_O_ is the initial height of the emulsion.

For thermal stability assessment, the same procedure was followed, except that the samples were incubated at 80 °C for 20 min before centrifugation.

### 2.8 Digital Microscope Analysis and Determination of Droplet Size Measurement of Mayonnaise

The microstructure and phase distribution of mayonnaise samples were analyzed using a digital optical microscope (VHX-7000, Keyence, Tokyo, Japan).^16^ Only mayonnaise samples exhibiting successful emulsification, namely the control, PO5SL3, and PO10SL2 formulations, were selected for detailed microscopic analysis and droplet size determination. These samples were considered representative of stable emulsions. Therefore, the droplet size analysis is limited to these formulations. A small aliquot of mayonnaise was placed on a glass microscope slide and covered with a coverslip before observation. The samples were first examined at 50× and 100× magnifications to observe the overall microstructure and phase distribution of the emulsions. Micrographs were captured using the Keyence imaging software.

Oil droplet diameters were measured directly from higher-magnification micrographs using Keyence image analysis software, with a minimum of 750 droplets analyzed per sample. To clearly resolve individual droplets, measurements were performed at 500× magnification for PO5SL3 and PO10SL2 samples and at 2000× magnification for the control sample, due to its smaller droplet size. As different magnifications were used, droplet size comparisons should be interpreted based on measured values and scale bars rather than visual appearance alone. The Sauter mean diameter (D­[3,2]) was calculated according to the following equation:

$$D\left[3,2\right]=\frac{\sum n_{i}{d_{i}}^{3}}{\sum {d_{i}}^{2}}$$

where n_i_ is the count of droplets with diameter d_i_ (μm).

The width of the droplet size distribution, known as the span, was calculated using this equation:

$$Span=\frac{d_{0.9}-d_{0.1}}{d_{0.5}}$$

where d_0.1_, d_0.5_, and d_0.9_ represent the droplet diameters (μm) below which 10%, 50%, and 90% of the total droplets are found, respectively.

### 2.9 Accelerated Oxidation Test

Only mayonnaise samples exhibiting successful emulsification, namely the control, PO5SL3, and PO10SL2 formulations, were selected for accelerated oxidation analysis. All samples were analyzed in triplicate. Accelerated oxidation tests were conducted to simulate long-term lipid oxidation at elevated temperatures. Mayonnaise samples were transferred into 250 mL Schott bottles and incubated in an oven at 60 °C. The samples were stored for 0, 24, and 72 h, after which lipid oxidation was evaluated. The oil fraction was extracted from the mayonnaise samples and subsequently analyzed for peroxide value (PV) and p-anisidine value (p-AV) to quantify primary and secondary oxidation products, respectively.^17^

### 2.10 Extraction of Mayonnaise Fat

A 50 g portion of the sample was placed into a centrifuge tube and frozen at −20 °C for 24 h to promote emulsion destabilization. After thawing at room temperature, the samples were centrifuged at 2,400 × *g* for 5 min. The separated oil layer was then collected and stored at −20 °C for subsequent analysis.^17^

### 2.11 Determination of Free Fatty Acid Content (FFA%)

The free fatty acid content (as oleic acid, %) of the pure and blended vegetable oils, as well as the lipid fraction from the prepared fat spreads, was determined. Samples were first dissolved in neutralized ethanol–diethyl ether (1:1, v/v) and then titrated with 0.1 M NaOH.^18^ All samples were analyzed in triplicate.

### 2.12 Determination of PV

Lipid hydroperoxide was measured as the PV of the mayonnaise samples. Fat was extracted, mixed with acetic acid:chloroform (3:2, v/v), and shaken. Potassium iodide (0.25 mL) was added to liberate iodine from peroxides. Then, 15 mL of water was added, and the iodine was titrated with 0.01 N Na_2_S_2_O_3_ using starch as an indicator. PV was calculated and reported as meq O_2_/kg. All samples were analyzed in triplicate.^18^

### 2.13 Determination of P-AV

1 g of extracted oil was dissolved in isooctane, and its absorbance at 350 nm was recorded. Then, 1 mL of p-anisidine reagent (in glacial acetic acid) was added, mixed, and the absorbance at 350 nm was remeasured. This assay quantifies aldehydic compounds, primarily 2-alkenals and 2,4-dienals, by reacting the acetic acid solution of aldehydes in the oil with p-anisidine and measuring absorbance at 350 nm. All samples were analyzed in triplicate.^18^

### 2.14 Statistical Analysis

All results were expressed as mean ± standard deviation. Unless otherwise stated, all analyses were performed using three replicate samples for each mayonnaise formulation. Statistical analysis was performed using SPSS software, employing one-way analysis of variance (ANOVA) followed by Tukey’s post hoc test to identify significant differences between groups. A significance level of *p* < 0.05 was considered statistically significant.

## 3 Results and Discussion

### 3.1 pH Values

Mayonnaise is a mildly acidic emulsion, and pH is critical for microbial stability and consumer acceptability, as undesirable levels compromise safety.^13,19^ It also influences emulsion stability by affecting egg yolk proteins, which form stabilizing networks of denatured molecules. Their effectiveness is greatest near the isoelectric point, where the net charge is minimized, and viscoelasticity is maximized.^19^ No significant differences in pH (*p* > 0.05) were observed among samples, with values ranging from 4.17 to 4.35 (Figure 1). These values were slightly higher than the typical commercial range (3.6–4.0),^20^ but remained within the broader range of 3.2–4.2.^3^ Notably, pH 4.3 is insufficient to inactivate Salmonella, and pH 4.2 was identified as the threshold for controlling *S. typhimurium*, with reduced temperatures further diminishing the effectiveness of acid.^19^ The control sample exhibited a pH of 4.35, indicating limited intrinsic acidity and a reduced ability to suppress acid-tolerant microorganisms, such as Salmonella. The other samples (pH 4.18–4.30) may offer slightly greater antimicrobial potential. Since pH can be adjusted with vinegar,^1^ the findings suggest that replacing egg yolk with RBD PO and SL could improve the pH profile of mayonnaise, enhancing microbial safety without compromising product quality. Further acidification could be achieved by controlled addition of food-grade acids (e.g., vinegar or citric acid) to the aqueous phase, thereby lowering pH while preserving lecithin-mediated interfacial stability.

**Figure 1:**
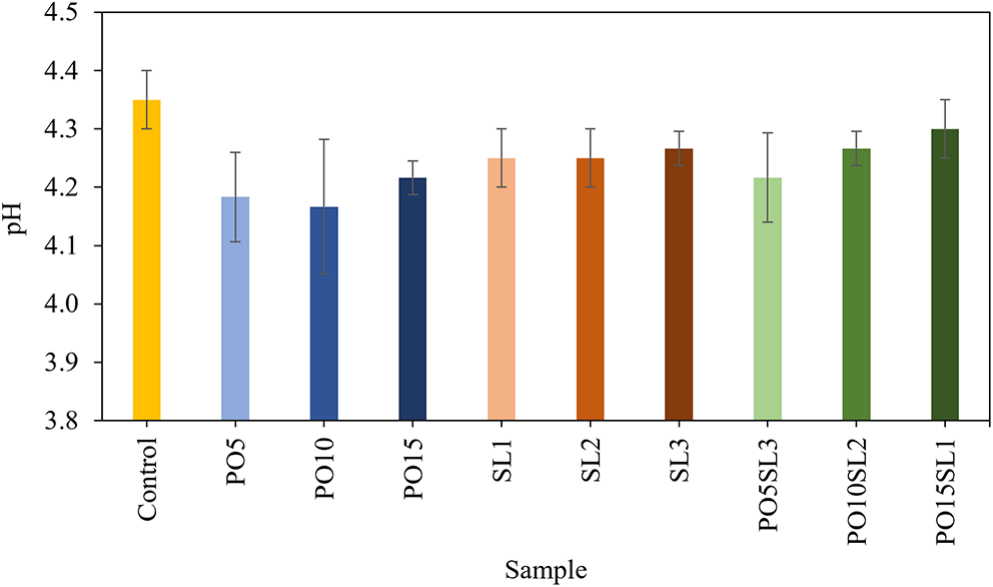
pH values of the mayonnaise samples. Results display mean ± standard deviation (*n* = 3) values. No significant differences in pH were observed among the samples (*p* > 0.05).

### 3.2 Effect on Water Activity

Water activity influences shelf life and microbial growth in foods.^19^ In this study, all vegan mayonnaise samples had water activity values of 0.98–0.99 (results not shown), with no significant differences (*p* > 0.05) among the formulations. These values, close to those of commercial mayonnaise (0.956),^14^ likely reflect consistent water binding by the main ingredients and the similar proportions of water-phase components such as boiled potatoes. Although the water activity range is favorable for microbial growth, no visible bacteria, mold, or yeast developed, probably due to the antimicrobial effects of vinegar, salts, and sugars.^21^ These findings confirm that using liquid SL and RBD PO as egg replacers does not affect the water activity of vegan mayonnaise. However, the relatively high water activity may pose a risk of microbial growth during extended storage. Therefore, appropriate preservation strategies, such as refrigeration, acidification, or the use of preservatives, are required to ensure long-term shelf stability.

### 3.3 Appearance and Color Parameters

Color strongly influences consumer perception, with mayonnaise typically associated with a light-yellow hue. Substituting conventional ingredients may alter color, thereby affecting acceptance and purchase decisions.^22^ While most formulations formed stable emulsions, samples PO5, PO10, PO15, and PO15SL1 exhibited phase separation (Figure 2). This instability is attributed to the properties of RBD PO. The refining process depletes natural emulsifiers (e.g., phospholipids and monoacylglycerols), thereby diminishing the oil’s emulsifying capacity.^23^ Furthermore, the high concentration of saturated, high-melting triacylglycerols (TAGs) in RBD PO may crystallize upon cooling, creating solid fat particles that can disrupt the oil–water interface and induce partial coalescence. This destabilization is exacerbated by RBD PO’s broad melting range and characteristic crystal morphology, which accelerate solid fat formation and phase separation.^6^

**Figure 2:**
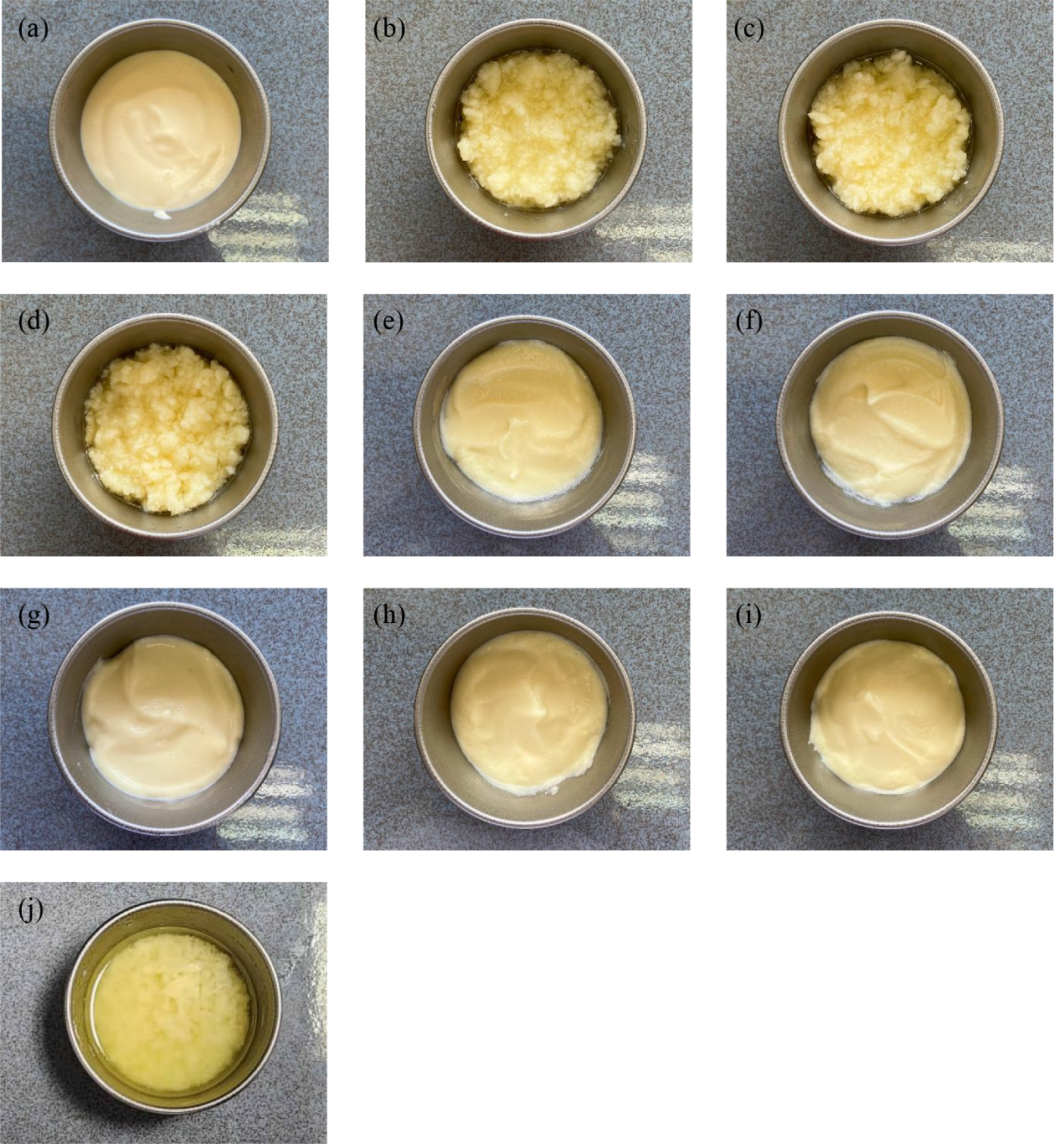
Visual appearance of mayonnaise samples. (a) Control, (b) PO5, (c) PO10, (d) PO15, (e) SL1, (f) SL2, (g) SL3, (h) PO5SL3, (i) PO10SL2, and (j) PO15SL1.

The colorimeter results (L*, a*, b*) are shown in Figure 3. L* differed significantly between the RBD PO group and the control (*p* < 0.05), while SL and PO–SL formulations did not differ significantly (*p* > 0.05). RBD PO–only formulations (PO5, PO10, PO15) showed lower L* (darker) than the control, due to the oil’s natural yellow color and its high-melting TAGs that increase light scattering; the effect intensified with higher RBD PO levels (PO10, PO15) via greater crystallization.^6,23^ The effect became more pronounced at higher RBD PO levels (PO10, PO15), where the increased extent of crystallization of high-melting TAGs may further intensify light scattering, impairing both visual appearance and stability. By contrast, PO–SL formulations maintained L* values comparable to (or slightly above) the control, with only minor decreases at higher SL, likely reflecting lecithin’s darker color.

**Figure 3:**
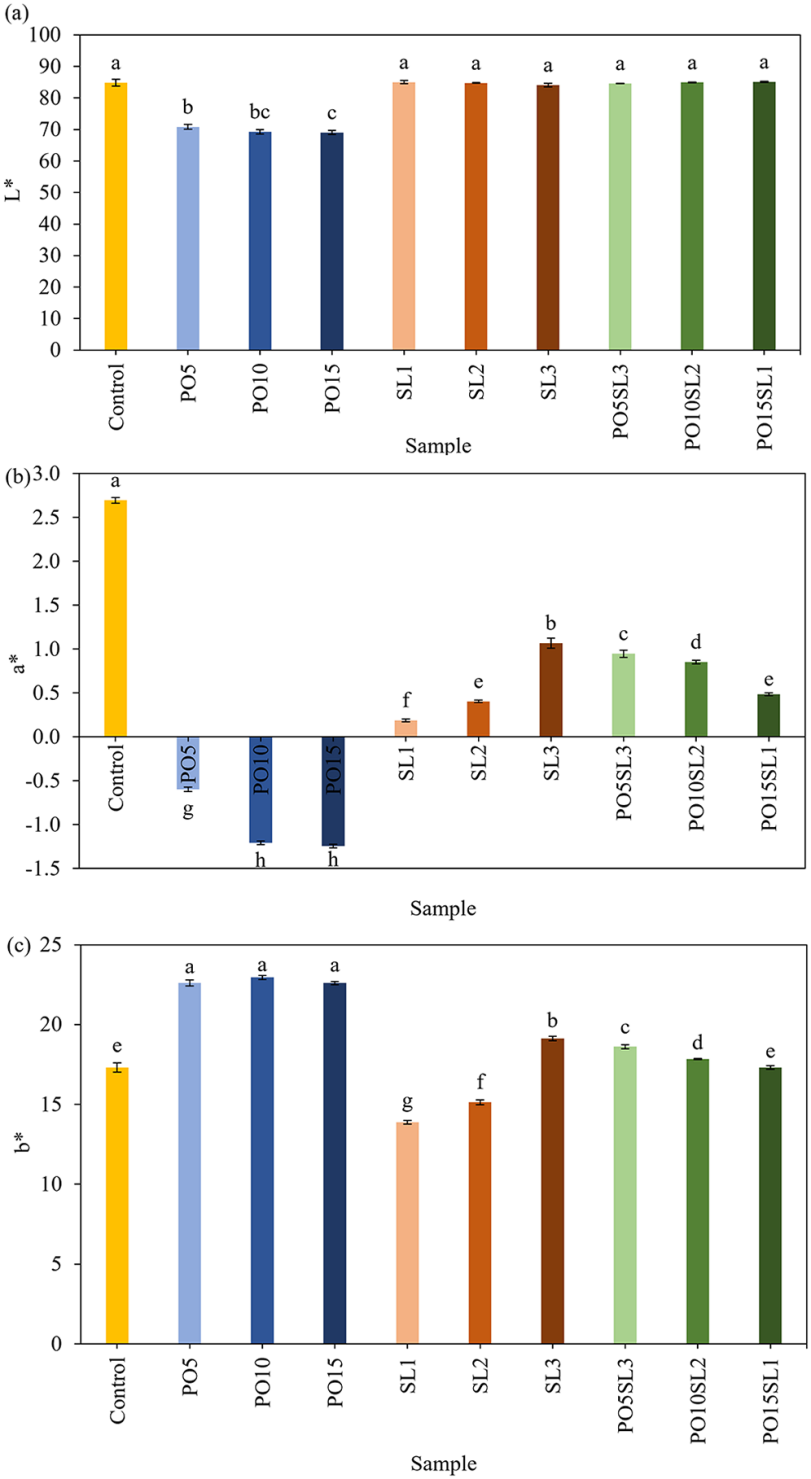
Color values of the mayonnaise samples. (a) Lightness, (b) redness, and (c) yellowness. Results display mean ± standard deviations (*n* = 3) values. Letters^a–h^ indicate significant differences (*p* < 0.05) between different bars.

The control (egg yolk) showed slightly positive a*, consistent with yolk carotenoids (lutein, zeaxanthin).^24^ RBD PO–only samples had a negative a* (greenish), reflecting carotenoid/tocotrienol losses during refining; the green tint increased with higher RBD PO.^23^ The SL formulations differed from control (*p* < 0.05), with higher a* as the SL level rose, attributable to its inherent brown pigments (carotenoids, flavonoids, Maillard products).^9^ However, in PO–SL formulations, a* decreased significantly (*p* < 0.05) because RBD PO masked SL pigment contributions, yielding lower net redness.

The control showed moderate yellowness typical of full-fat mayonnaise.^16^ SL formulations exhibited a significant increase in b* with higher SL levels (*p* < 0.05), which is linked to carotenoids and Maillard products.^9^ In PO–SL formulations, b* decreased with increasing RBD PO (*p* < 0.05), reflecting the lighter yellow of refined oil.^23^ Nevertheless, the SL and PO–SL formulations had b* values comparable to those of the control (*p* > 0.05), suggesting that SL chiefly enhances color uniformity rather than imposing an intense color. All samples maintained an acceptable visual appearance.

### 3.4 Textural Properties

Figure 4 presents the textural properties of the mayonnaise samples, where firmness reflects resistance to probe penetration, with higher values indicating greater resistance to deformation.^25^ The control exhibited the lowest firmness (*p* < 0.05), consistent with the strong emulsifying capacity of egg yolk, which produces finely dispersed droplets and a flexible interfacial network capable of accommodating deformation.^26^ In contrast, formulations containing RBD PO showed significantly higher firmness (*p* < 0.05), which increased with oil content. This behavior can be attributed to the semisolid nature and higher saturated fatty acid content of RBD PO, which may promote droplet flocculation and partial coalescence, likely associated with partial crystallization of saturated TAGs. Similar trends have been reported for emulsions formulated with saturated-fat-rich oils, highlighting the strong influence of lipid composition on mayonnaise texture.^25,27^

**Figure 4:**
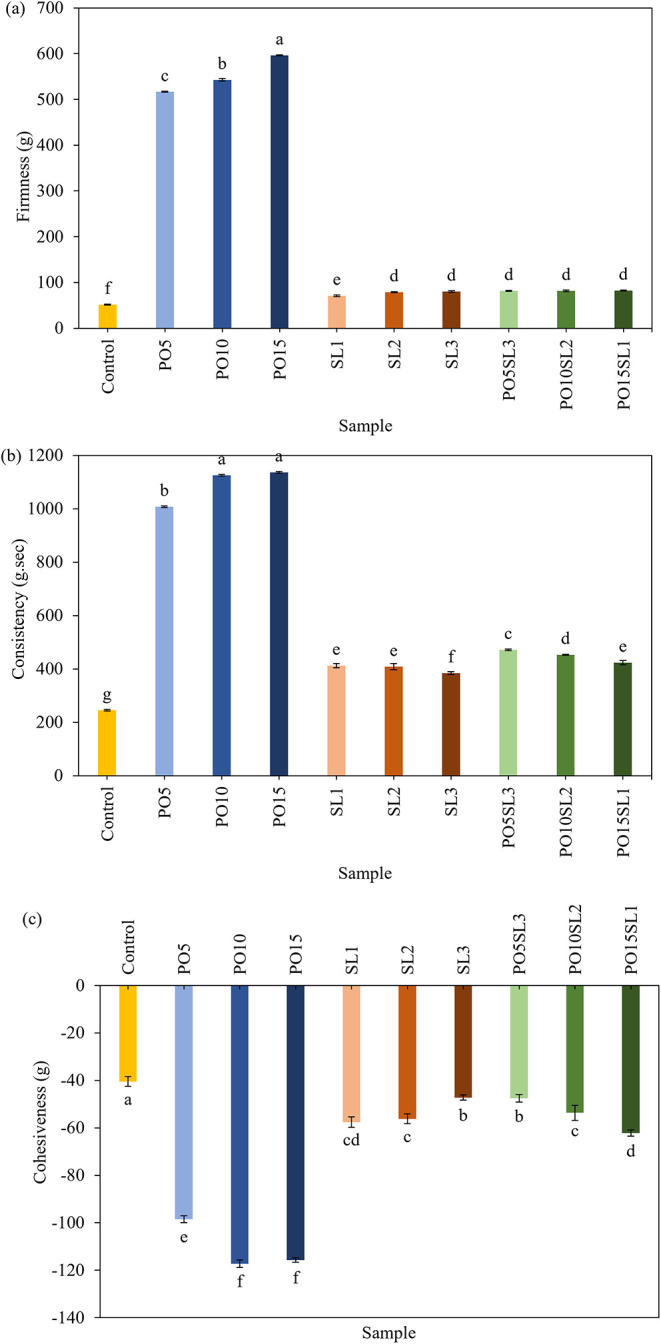
Textural properties of mayonnaise samples. (a) Firmness, (b) consistency, and (c) cohesiveness. Results display mean ± standard deviations (*n* = 3) values. Letters^a‑g^ indicate significant differences (*p* < 0.05) between different bars.

Both SL and PO–SL formulations exhibited significantly higher firmness than the control (*p* < 0.05), which can be attributed to the strong emulsifying capacity of sunflower lecithin and the efficient interfacial packing of its phospholipid components (PC, PE, PI, and PA). These phospholipids form a cohesive and viscoelastic interfacial film that enhances droplet stability and promotes the development of a dense, gel-like microstructure, thereby increasing resistance to deformation.^8^ At higher SL concentrations, firmness approached a plateau, indicating interfacial saturation, in which droplet surfaces are fully covered and additional lecithin primarily reduces droplet size rather than further strengthening the network. This interpretation is supported by the droplet size distribution data (Table 2) and microstructural observations (Figure 7 and Figure S1), which demonstrate smaller and more uniformly distributed droplets in lecithin-containing formulations. Consequently, the system appears to transition from interfacial reinforcement to steric stabilization, limiting further increases in rigidity.

**Table 2:** Oil Droplet Size Distribution of the Mayonnaise Sample^a^

Sample	D [3,2] (μm)	Span
Control	1.53 ± 0.24^b^	0.564 ± 0.144^b^
PO5SL3	5.81 ± 0.79^b^	0.572 ± 0.123^b^
PO10SL2	23.8 ± 5.24^a^	1.568 ± 0.213^a^

a Results display mean ± standard deviations (*n* = 750) values. Letters^a–b^ indicate significant differences (*p* < 0.05) between different columns.

Consistency followed trends similar to firmness. RBD PO formulations exhibited significantly higher consistency than the control (*p* < 0.05), likely due to partial droplet coalescence and the formation of a percolated fat crystal network that enhances rigidity and resistance to flow, as commonly observed in emulsions containing crystallizing lipid phases.^28^ Consistency increased markedly at 5% RBD PO and then plateaued between 5% and 10%, suggesting structural saturation of the emulsion matrix. The control sample showed the lowest consistency, reflecting a more homogeneous droplet distribution and limited structural networking.

SL and PO–SL formulations also exhibited higher consistency than the control (*p* < 0.05), which can be attributed to lecithin-mediated reductions in droplet size and strengthening of the interfacial film, thereby suppressing coalescence and enhancing bulk viscosity.^29^ However, at higher SL levels, consistency decreased, consistent with an overstabilization effect in which excessive emulsifier produces smaller, more uniformly dispersed droplets with reduced droplet–droplet interactions, resulting in a weaker continuous network and lower apparent body.^30^ This behavior suggests a functional limit to lecithin inclusion, where excessive emulsifier reduces structural interactions, and also indicates a practical/economic limit, as higher lecithin levels do not provide additional textural benefits. A similar reduction in consistency was observed in PO–SL formulations at higher RBD PO levels, likely due to the higher proportion of unsaturated lipids, which soften the droplet matrix and reduce structural rigidity, as previously reported for mayonnaise formulated with virgin red palm oil.^11^ In such systems, the incorporation of hydrocolloids such as modified maize starch or xanthan gum may be required to restore desirable texture, as demonstrated in reduced-fat mayonnaise formulations.^31^

Cohesiveness exhibited an inverse relationship with firmness, with lower values indicating a more brittle, less recoverable structure.^25^ RBD PO formulations displayed the lowest cohesiveness (*p* < 0.05), consistent with emulsion instability arising from fat crystallization and insufficient interfacial stabilization.^32^ Although higher RBD PO levels increased firmness, they produced a rigid yet weakly connected droplet network, leading to poor structural recovery after deformation. SL and PO–SL formulations also showed reduced cohesiveness compared to the control (*p* < 0.05), likely due to the formation of well-separated droplets that limit interdroplet bonding. At elevated SL concentrations, excess unadsorbed lecithin in the continuous phase may further act as a lubricant or induce interfacial crowding, thereby reducing structural recovery.

Overall, the observed textural differences reflect variations in interfacial film strength and fat crystal behavior. In RBD PO–rich systems, the presence of high-melting saturated triacylglycerols promotes partial crystallization within oil droplets, leading to increased firmness but reduced cohesiveness. Conversely, SL enhances interfacial elasticity through dense phospholipid packing, improving droplet dispersion and resistance to deformation. These findings highlight the dominant role of interfacial chemistry, rather than bulk composition alone, in determining the mechanical behavior of concentrated food emulsions.

### 3.5 Viscosity Values

Viscosity differed significantly among formulations (*p* < 0.05), highlighting the strong influence of formulation composition on emulsion flow behavior (Figure 5). The control sample exhibited higher viscosity than the RBD PO formulations but lower viscosity than both SL and PO–SL formulations (*p* < 0.05). The increased viscosity observed in SL-containing systems can be attributed to reduced droplet size and enhanced interfacial film strength, which increase droplet surface area and interdroplet friction under shear. In contrast, RBD PO formulations displayed the lowest viscosities, likely due to insufficient interfacial stabilization and the onset of partial phase separation, as RBD PO lacks intrinsic surface-active components capable of maintaining a fine emulsion structure. These findings are consistent with previous observations that viscosity reflects not only bulk composition but also interfacial organization and droplet interactions, and therefore correlates only partially with the texture profile parameter.^25^

**Figure 5:**
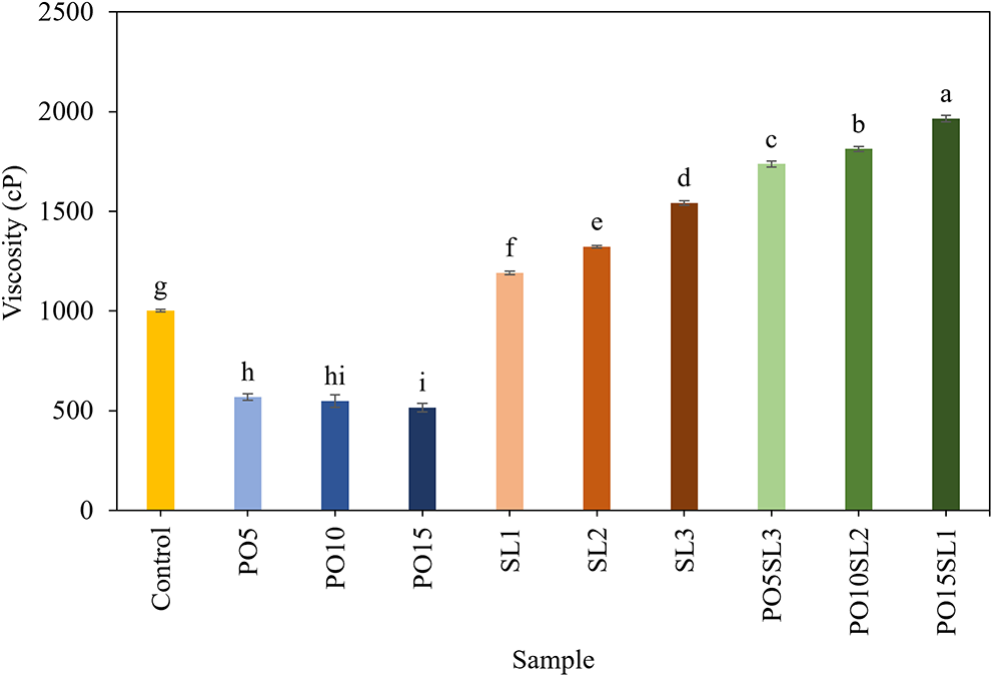
Viscosity of mayonnaise samples. Results display mean ± standard deviations (*n* = 3) values. Letters^a–i^ indicate significant differences (*p* < 0.05) between different bars.

Among formulations, PO–SL systems exhibited the highest viscosities, with PO15SL1 showing a significantly greater value than the others (*p* < 0.05). This behavior reflects the combined roles of lecithin and RBD PO. Lecithin stabilizes the emulsion through interfacial adsorption and droplet size reduction, while RBD PO contributes to bulk structuring and viscosity, likely associated with its saturated triacylglycerol content and partial crystallization behavior. However, RBD PO alone does not form stable emulsions, indicating that lecithin acts as the primary stabilizing agent while palm oil plays a secondary role in modulating rheological properties.^8,29,34^ In addition, the inclusion of boiled potato contributes to structuring the continuous phase of the emulsion. The starch present in the potato can act as a bulk viscosity modifier, increasing the viscosity of the aqueous phase and potentially reducing droplet mobility and creaming behavior. However, as the potato content was kept constant across all formulations, its effect is considered uniform. Therefore, while starch-induced viscosity contributes to baseline emulsion structure, the observed differences in microstructure, rheology, and oxidative behavior are primarily attributable to variations in SL and RBD PO. While potato contributes to baseline viscosity, it is unlikely to confound comparative interpretation across formulations. Overall, viscosity is likely governed by the combined contributions of interfacial stabilization and bulk structuring, although a direct correlation between lecithin surface coverage and oil surface area was not established.

### 3.6 Emulsification Stability

Emulsification stability, as quantified by the CI, showed significant differences among formulations (*p* < 0.05), where CI = 0 indicates complete stability.^35^ As shown in Figure 6, significant differences were observed among formulations (*p* < 0.05). The lowest CI values, indicating superior stability, were recorded for the control and SL-containing formulations, whereas RBD PO–only samples exhibited significantly higher CI values. No significant differences were observed between SL and PO–SL formulations (*p* > 0.05), indicating that lecithin acts as the primary stabilizing agent. Visual observations after centrifugation indicated that destabilization occurred primarily through droplet creaming rather than complete oil separation in the control and lecithin-containing samples. These samples formed a cream layer at the top of the tube while the emulsion structure remained largely intact, suggesting effective interfacial stabilization. In contrast, the RBD PO–only samples exhibited visible free oil separation, indicating partial emulsion breakdown and weaker interfacial stabilization. This behavior is consistent with the higher CI values observed for these samples and aligns with the visual phase separation reported in Figure 2 (Section 3.3), suggesting reduced emulsion stability in RBD PO–only formulations.

**Figure 6:**
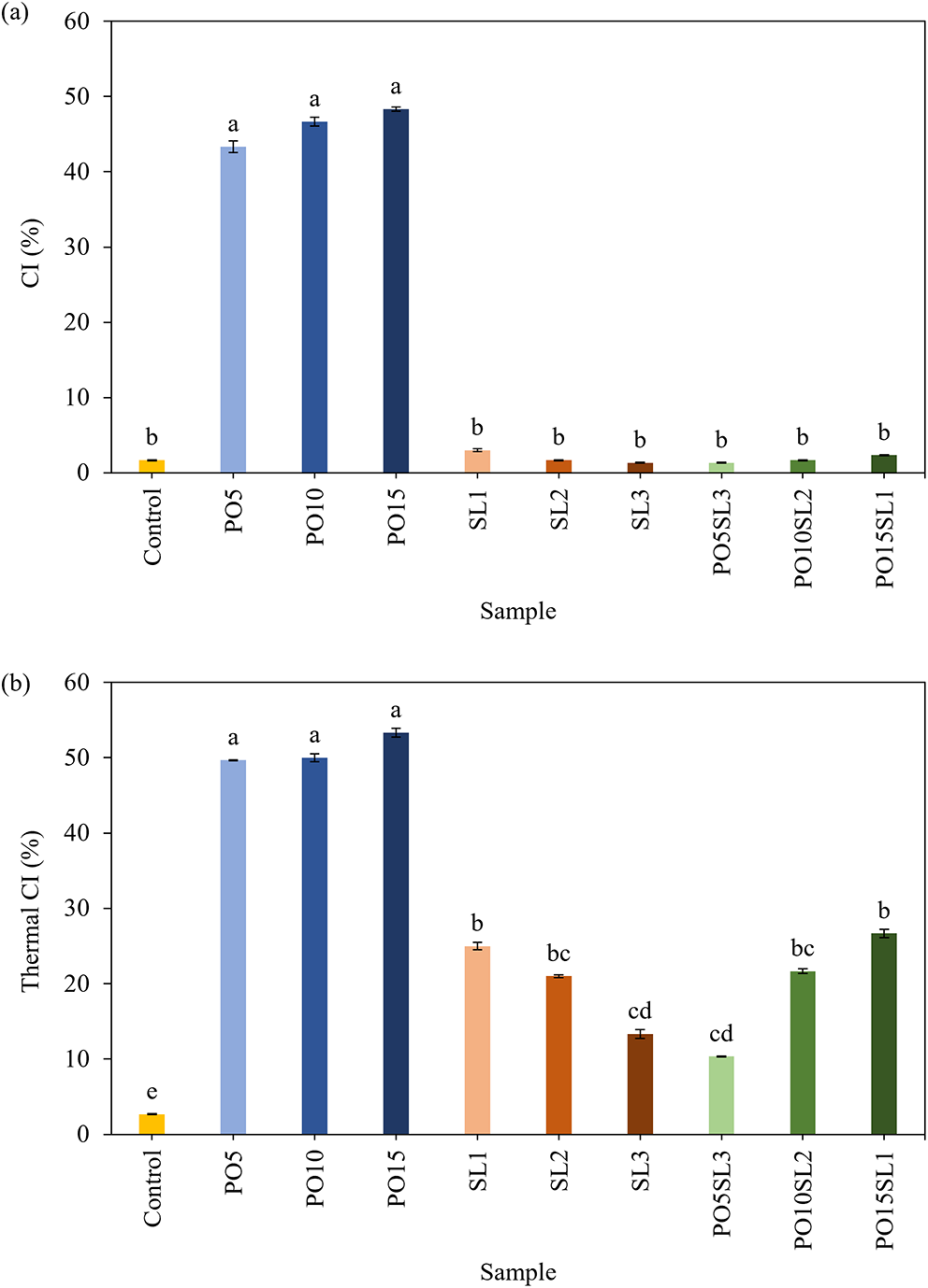
(a) CI and (b) thermal CI of mayonnaise samples. Results display mean ± standard deviations (*n* = 3) values. Letters^a–e^ indicate significant differences (*p* < 0.05) between different bars.

The superior stability of the control sample can be attributed to the presence of egg-yolk phospholipids and lipoproteins, which form robust interfacial films that resist droplet coalescence and thermal disruption.^36^ In contrast, RBD PO formulations exhibited higher CI values (*p* < 0.05), reflecting poorer stability due to their limited emulsifying capacity. Increasing RBD PO further exacerbated instability, particularly under thermal conditions.^37^ This behavior arises because RBD PO consists predominantly of triacylglycerols with minimal surface-active components, relying mainly on mechanical homogenization and fat-crystal structuring rather than interfacial stabilization.^6^ Under heating, the melting of lower-melting TAG fractions weakens the interfacial layer, increases droplet mobility, and promotes coalescence, consistent with the behavior reported for palm oil-based emulsions.^38^

The incorporation of SL significantly improved emulsion stability by enhancing interfacial coverage and reducing oil–water interfacial tension. Phospholipids, such as lecithin, rapidly adsorb at the droplet surface, forming a viscoelastic interfacial layer that provides steric and electrostatic repulsion, thereby limiting droplet aggregation and creaming.^33^ This indicates that emulsion stability is primarily governed by interfacial stabilization rather than by bulk composition. However, as the proportion of RBD PO increased in PO–SL systems, thermal creaming became more pronounced. This effect is likely associated with partial displacement of lecithin molecules from the interface by TAGs that may undergo partial crystallization, thereby disrupting interfacial continuity and weakening stabilizing forces under heat stress. In addition, minor nonlipid components in lecithin, such as residual proteins or ash, may further reduce interfacial stability at elevated temperatures.^39^

Despite these effects, both SL and PO–SL formulations exhibited CI values comparable to the control under nonthermal conditions, indicating effective stabilization of the oil–water interface. While lecithin is essential for interfacial stabilization, RBD PO contributes to bulk structuring through its saturated triacylglycerol content, influencing viscosity and droplet rigidity, likely through partial crystallization. However, RBD PO alone is insufficient to maintain emulsion stability, indicating that its role is secondary to that of lecithin. Based on these mechanistic considerations, PO5SL3 and PO10SL2 were selected for further analysis, as they exhibited the most favorable balance between stability and formulation performance.

### 3.7 Digital Microscope Analysis and Oil Droplet Size Distribution

Figure 7 shows the micrographs of control, PO5SL3, and PO10SL2 samples. The digital microscope images demonstrated that all samples were oil-in-water emulsions, characterized by oil droplets dispersed throughout the continuous aqueous phase. However, clear differences in droplet size were observed. The control and PO5SL3 samples exhibited fairly consistent oil droplet sizes, whereas PO10SL2 showed a wider range of droplet diameters. Additionally, PO10SL2 displayed a porous structure with numerous gaps or interstitial areas, which likely correspond to regions occupied by the aqueous phase or trapped air bubbles within the emulsion.^16^

**Figure 7:**
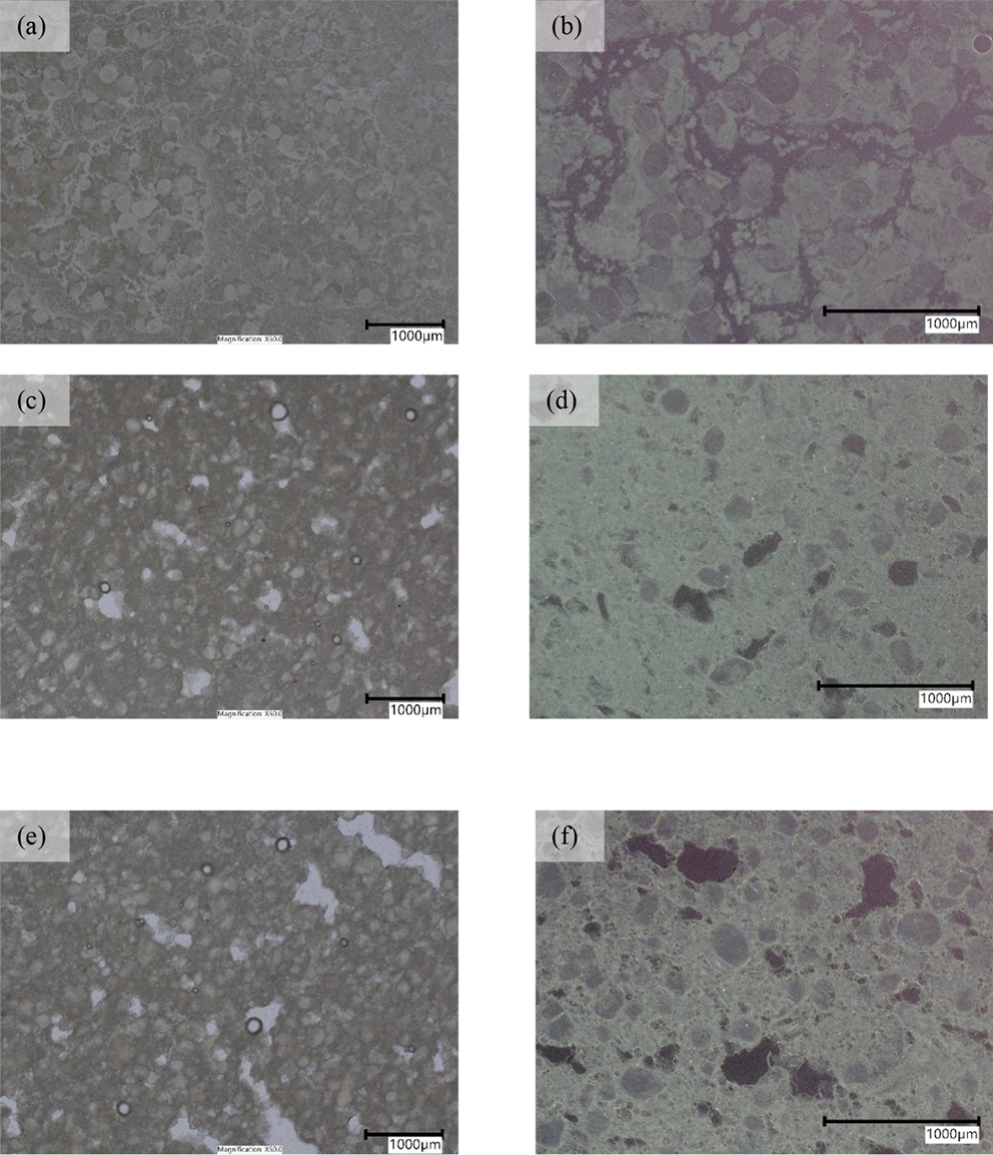
Micrographs of mayonnaise samples at 50× and 100× magnifications, respectively. (a) and (b) control, (c) and (d) PO5SL3, and (e) and (f) PO10SL2.

The Sauter mean diameter (D­[3,2]) and span (Table 2) were used to assess droplet size distribution. Representative higher-magnification micrographs used for droplet size measurements are provided in the (Supporting Information Figure S1). Droplet size comparisons are based on measured values (D­[3,2]) rather than visual appearance alone, particularly given the differing magnifications used. Control and PO5SL3 showed significantly (*p* < 0.05) lower D­[3,2] than PO10SL2, with no difference between control and PO5SL3 (*p* > 0.05). The control had the smallest droplets (1.53 μm), consistent with efficient emulsification and stabilization by egg-yolk components (coalesced LDL granules and phosphoproteins acting as interfacial barriers).^25^ In contrast, PO5SL3 and PO10SL2 had average droplet sizes of 5.81 and 23.8 μm, respectively. These findings for the selected formulations suggest that increasing the RBD PO while lowering SL content increases oil droplet size by reducing emulsion stability and interfacial stability. In a concentrated emulsion, such as mayonnaise, this promotes coalescence and the formation of larger droplets.^25^ This is consistent with prior studies showing that higher lecithin levels produce smaller droplets and more stable emulsions.^9^ With appropriate adjustments to the SL content and the RBD PO proportion, a droplet-size distribution comparable to that of the control should be attainable. However, this interpretation is limited to the formulations analyzed in this study. A quantitative correlation between lecithin concentration and oil surface area (derived from droplet size and volume fraction) was not established in this study, as droplet size analysis was limited to selected formulations. However, the results suggest that lecithin primarily influences interfacial stabilization through surface coverage, whereas oil composition contributes to bulk viscosity and structuring. Therefore, the formulations reported here should be viewed as the best within the studied window, rather than definitive optima.

An increase in RBD PO substitution coupled with reduced SL raised span, indicating a broader, more heterogeneous droplet-size distribution. The control (span = 1) showed excellent uniformity, and PO5SL3 also maintained a span slightly <1, comparable to that of the control. In contrast, PO10SL2 exhibited the highest span (1.568, *p* < 0.05), indicating marked polydispersity. Such a wide range of droplet sizes is critical because it directly affects texture, consistency, and stability.^16^ Larger and more variable droplets are more prone to aggregation and coalescence, resulting in a rougher texture and reduced quality.^33^ Lecithin mitigates these effects in the analyzed formulations by lowering interfacial tension and forming protective interfacial films that provide steric/electrostatic repulsion, thereby decreasing droplet size and polydispersity and promoting a uniform, stable distribution.^33^

### 3.8 FFA%

The oxidation of fatty acids may destabilize mayonnaise, generating off-flavors and odors, reducing shelf life, safety, and consumer acceptance.^16^ In this study, the FFA% increased significantly (*p* < 0.05) during storage across all formulations (Table 3). This rise is driven primarily by lipolysis, the hydrolysis of TAGs and phospholipids by lipases/phospholipases. At the same time, advanced oxidation can secondarily form low-molecular-weight acids that elevate the acid value.^2^ PO5SL3 and PO10SL2 exhibited higher FFA% than the control at each time point, with no significant difference between them (*p* > 0.05). This pattern reflects not only enhanced interfacial accessibility for lipolysis in emulsions but also lecithin’s intrinsic acidity, which inflates the titrimetric FFA%. In the control, part of the measured FFA% reflects the baseline acidity of egg yolk lipids, but this baseline is lower than the contribution from SL in PO5SL3/PO10SL2.^2^ Consistent with industry standards, the PORAM Trading Specification for Processed Palm Oil limits RBD PO to ≤0.1% FFA,^40^ while international specifications for lecithin set AV ≤ 35 mg KOH/g (≤45 mg KOH/g for hydrolyzed lecithin) and PV < 10 mequiv O_2_/kg.^9^ FFAs act as prooxidants, surface-active impurities, and chelating agents, accelerating hydroperoxide decomposition and lowering interfacial tension to facilitate oxygen transfer.^41^ Therefore, keeping base-oil FFA low limits prooxidant effects, while the higher FFA % in lecithin formulations mainly reflects lipolysis and the intrinsic acidity of lecithin, rather than ongoing oxidation.

**Table 3:** FFA, PV, and P-AV of Selected Mayonnaise Formulations over Storage at 60 °C^a^

Oxidation parameter	Storage time (h)	Control	PO5SL3	PO10SL2
PV (meq/kg)	0	1.86 ± 0.18^Ca^	1.45 ± 0.23^Cb^	1.33 ± 0.23^Cb^
	24	3.13 ± 0.44^Ba^	2.78 ± 0.37^Bb^	2.65 ± 0.24^Bb^
	72	4.94 ± 0.27^Aa^	4.10 ± 0.24^Ab^	3.95 ± 0.41^Ab^
p-AV (g/100 mL)	0	1.33 ± 0.29^Ca^	0.88 ± 0.09^Cb^	0.80 ± 0.20^Cb^
	24	1.98 ± 0.08^Ba^	1.56 ± 0.11^Bb^	1.45 ± 0.10^Bb^
	72	2.81 ± 0.21^Aa^	2.23 ± 0.26^Ab^	2.15 ± 0.08^Ab^
FFA (%)	0	0.46 ± 0.06^Cb^	2.31 ± 0.05^Ca^	2.24 ± 0.1B^Ca^
	24	0.88 ± 0.12^Bb^	2.66 ± 0.05^Ba^	2.57 ± 0.06^Ba^
	72	1.26 ± 0.16^Ab^	3.09 ± 0.16^Aa^	2.97 ± 0.07^Aa^

a Results display mean ± standard deviation (*n* = 3) values. Letters^A–C^ indicate significant differences (*p* < 0.05) between storage times in the same sample. Letters^a–b^ indicate significant differences (*p* < 0.05) between samples in the same row.

### 3.9 Oxidation

PV served as an index of primary oxidation (peroxides and hydroperoxides), where lower values indicate reduced oxidation and improved stability.^42^ As shown in Table 3, PV and p-AV increased significantly during storage for all samples (*p* < 0.05), consistent with the formation of hydroperoxides and their subsequent decomposition into secondary carbonyl compounds, a process known to be accelerated at the oil–water interface.^2^ The control exhibited significantly higher PV and p-AV values than the PO–SL formulations (*p* < 0.05). This behavior can be attributed to the oxidation of egg-yolk lipids during processing, which compromises emulsifying capacity and promotes droplet coalescence, leading to less stable emulsions with larger droplet sizes.^36^ In contrast, the lower PV and p-AV values observed in PO5SL3 and PO10SL2 reflect the higher intrinsic oxidative stability of RBD PO^7^ and the protective interfacial barrier formed by sunflower lecithin, which limits oxygen diffusion and stabilizes the droplet interface.^10^

Notably, lecithin contributes to oxidative stability primarily through interfacial and microstructural effects rather than direct antioxidant activity.^9^ Its ability to form a compact phospholipid layer at the oil–water interface restricts oxygen access and retards hydroperoxide decomposition. However, its antioxidative performance is strongly formulation- and concentration-dependent. In certain systems, lecithin has been reported to attenuate the activity of phenolic antioxidants and exhibit limited synergism with α-tocopherol, highlighting the importance of optimizing its concentration for specific formulations.^34^ The consistently lower oxidative indices observed in PO–SL formulations therefore support the central role of interfacial structuring in controlling lipid oxidation. Phospholipid adsorption at the oil–water interface acts as a physical diffusion barrier, reducing oxygen permeability and delaying the formation and breakdown of hydroperoxides. These findings demonstrate that improved oxidative stability in egg-free mayonnaise arises predominantly from interfacial stabilization mechanisms rather than from intrinsic antioxidant capacity alone.

## 4 Conclusion

This study demonstrates that the interplay between lipid composition and interfacial stabilization governs the physicochemical and oxidative stability of egg-free mayonnaise. SL acts as the primary stabilizing agent through phospholipid-mediated interfacial adsorption, while RBD PO contributes to bulk structuring and textural properties, likely associated with its saturated triacylglycerol content. The balance between these components influences droplet characteristics, rheological behavior, and oxidative stability. Among the formulations evaluated, PO5SL3 exhibited the most favorable combination of structural integrity, emulsion stability, and oxidative resistance. These findings highlight the importance of interfacial chemistry in determining emulsion behavior, with bulk lipid properties playing a secondary role in modulating texture. The results provide mechanistic insight into lipid–emulsifier interactions in egg-free systems and support the development of stable plant-based mayonnaise formulations. However, these findings should be interpreted within the formulation window investigated, particularly with respect to droplet size. Further work is required to relate emulsifier concentration to interfacial surface area quantitatively and to better distinguish interfacial and bulk contributions to emulsion behavior.

## Supplementary Material



## Data Availability

The data supporting the findings of this study are not publicly available due to their relevance to ongoing and unpublished work, but are available from the corresponding author upon reasonable request.

## Notes

Figure S1: micrographs of mayonnaise samples used for droplet size measurement. The control sample was observed at 2000× magnification, whereas PO5SL3 and PO10SL2 were observed at 500× magnification. Comparisons should be made using the scale bars, as differing magnifications may affect the apparent droplet size. (a) Control, (b) PO5SL3, and (c) PO10SL2 (PDF)
