# Supplementary material for: The Impact of Refined, Bleached, and Deodorized Palm Oil and Sunflower Lecithin on the Physicochemical Properties and Oxidative Stability of Egg-Free Mayonnaise
Source: ACS Omega. 2026 Jun 9;11(24):35518–28. doi: 10.1021/acsomega.6c00909 (PMC13295027; doi:10.1021/acsomega.6c00909)
Supplement: Supplementary file 1 [file ao6c00909_si_001.pdf]

The impact of refined, bleached, and deodorised palm oil and sunflower lecithin on the physicochemical properties and oxidative stability of egg-free mayonnaise

Shin-Yong Yeoh[1*], Nur Iesya Safiah Abdul Hamid[1], Lubowa Muhammad[2], Ahmad Syahir Zulkipli[3], Faheem Ullah[4], Hui-Ling Tan[5], Thuan-Chew Tan[1], Azhar Mat Easa[1]*

[1]Food Technology Division, School of Industrial Technology, Building G07, Persiaran Sains, Universiti Sains Malaysia, 11800 Pulau Pinang, Malaysia.

[2]Department of Food Innovation and Nutrition, Faculty of Agriculture and Environmental Sciences, Mountains of the Moon University, Fort Portal, Uganda.

[3]Earth Material Characterisation Laboratory, Centre for Global Archaeological Research, Universiti Sains Malaysia, 11800 Pulau Pinang, Malaysia.

[4]Bioresource Technology Division, School of Industrial Technology, Building G07, Persiaran Sains, Universiti Sains Malaysia, 11800, Pulau Pinang, Malaysia.

[5]School of Science, Monash University Malaysia, Jalan Lagoon Selatan, 47500 Bandar Sunway, Selangor Darul Ehsan, Malaysia.

*Corresponding authors: syongyeoh@gmail.com, syyeoh@usm.my (Shin-Yong Yeoh), azhar@usm.my (Azhar Mat Easa).

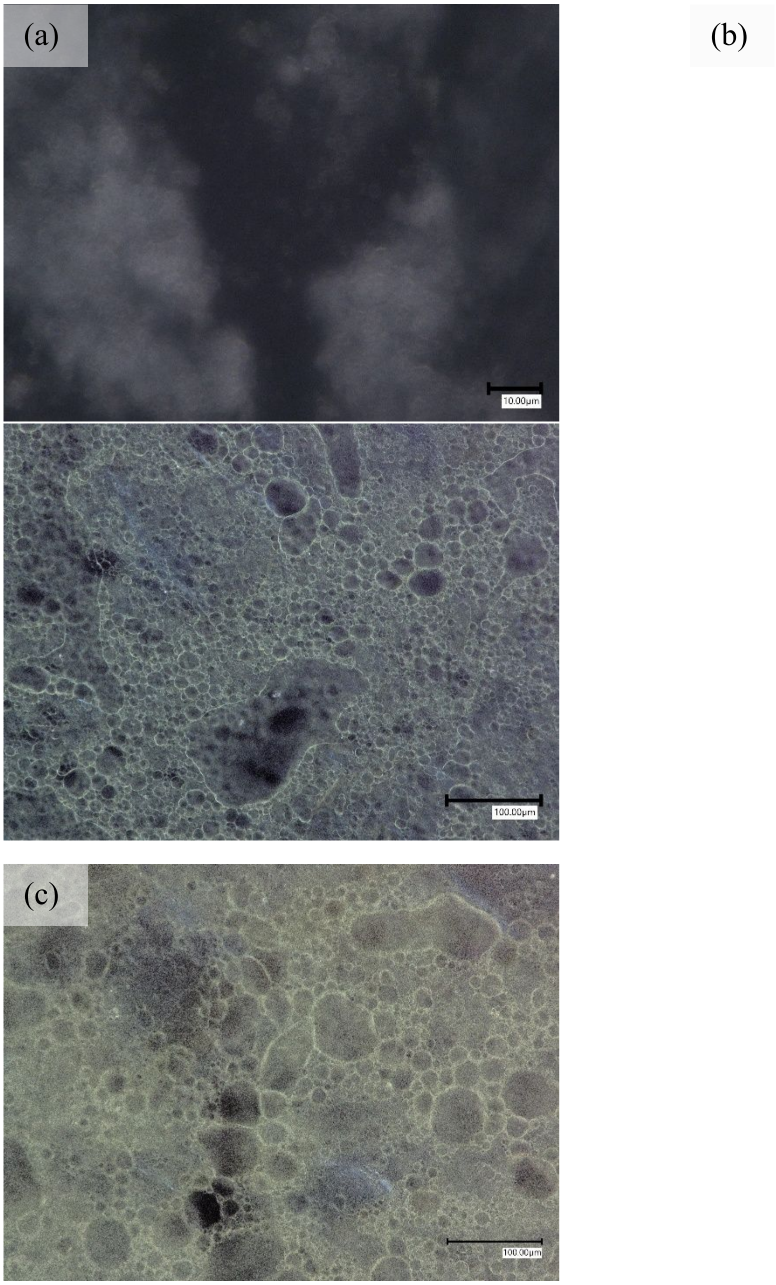

Figure S1: Micrographs of mayonnaise samples used for droplet size measurement. The control sample was observed at 2000× magnification, whereas PO5SL3 and PO10SL2 were observed at 500× magnification. Comparisons should be made using the scale bars, as differing magnifications may affect the apparent droplet size. (a) Control, (b) PO5SL3, and (c) PO10SL2.
